# Identifying pyroptosis-related lncRNAs to predict prognosis and immune regulation in hepatocellular carcinoma

**DOI:** 10.3389/fmolb.2026.1714966

**Published:** 2026-02-25

**Authors:** Wenjie Zhang, Yinjie Wang, Qiang Meng, Zhengcai Liu, Shibin Qu, Jingshi Zhou

**Affiliations:** 1 College of Life Sciences, Northwest University, Xi’an, Shaanxi, China; 2 Department of Hepatobiliary Surgery, Xijing Hospital, Fourth Military Medical University, Xi’an, Shaanxi, China; 3 Basic Medicine School, Fourth Military Medical University, Xi’an, Shaanxi, China

**Keywords:** hepatocellular carcinoma, LINC00942, pyroptosis-related long non-coding RNA, pyroptosis, risk score

## Abstract

**Background:**

Immunotherapy is often effective for treating hepatocellular carcinoma (HCC) and is widely used clinically, with variable effectiveness among individuals.

**Purpose:**

In this study, we aimed to develop a predictive signature from eight pyroptosis-related long non-coding RNAs (PRlncRNAs) for survival forecasting in HCC patients and immunotherapy evaluations.

**Methods:**

To assess the prognostic performance of the risk signature, we performed the following analyses: Kaplan–Meier survival analysis, receiver operator characteristics analysis, and nomogram construction.

**Results:**

The results showed that individuals within the high-risk cohort exhibited unfavorable prognosis, with the risk score acting as an independent predictor of prognosis. The PRlncRNAs in the high-risk cohort were significantly gathered in the immune-related signaling pathways; gene set enrichment analysis (GSEA) and single-sample GSEA showed that their functional activities were reliant on the immune cells, inhibitory immune checkpoints, and cytokines. Furthermore, we confirmed that downregulation of LINC00942 (a risk factor) expression increased pyroptosis levels and promoted infiltration of CD3 in HCC, while si-AC009283.1 group (a protective factor) exerted opposite effects *in vitro*.

**Conclusion:**

This risk signature developed using eight PRlncRNAs exhibits high reliability for predicting the prognosis of HCC patients and assessing their immune status, emphasizing their crucial roles in guiding immunotherapy and crafting precise treatments for the benefit of patients.

## 1 Introduction

Hepatocellular carcinoma (HCC) is a very common type of liver cancer and is the fourth most prominent cause of cancer-related deaths worldwide (Donne and Lujambio, 2023). Most patients are diagnosed with HCC at advanced stages of the disease, for which therapeutic options are frequently limited (Xing et al., 2021). Although immunotherapy, particularly the use of immune checkpoint inhibitors (ICIs) as monotherapy, have shown promise in the treatment of HCC, the effectiveness for patients in clinical settings in known to be only 15%–20%. This underscores the necessity to enhance the efficacy of immunotherapy (Rimassa et al., 2023). The NLR family pyrin domain-containing 3 (NLRP3) inflammasome-mediated pyroptosis is a key component of the NLRP3–ASC–caspase-1 complex and represents a specific type of programmed cell death. When endogenous danger signals or environmental stimuli occur, the cells may activate NLRP3/caspase-1 signaling and induce pyroptosis (Huang et al., 2021). Pyroptosis within cancer cells is triggered by the secretion of cytokines (IL-18 and IL-1β) and other inflammasomes, which could facilitate the activation and migration of immune cells, potentially leading to tumor reduction to some extent. Thus, pyroptosis induced by tumor cells can inhibit tumor growth and stimulate antitumor immunity, making it a potential treatment strategy for cancer (Rao et al., 2022). However, limited understanding regarding the pyroptosis mechanisms in HCC hinders its clinical application.

Long non-coding RNAs (lncRNAs) play crucial roles in various tumor biological processes and function as sponges, decoys, or scaffolds (Yan and Bu, 2021). Additionally, they are capable of regulating pyroptosis to influence tumorigenesis and immune responses (He et al., 2020). LncRNAs affect the incidence of pyroptosis, impact the prognosis of cancer patients, and influence immune reactions by regulating the NLRP3/caspase-1/GSDMD pathway (Yang et al., 2023). For instance, cisplatin treatment has been shown to trigger the MEG3-mediated pyroptosis pathway in triple-negative breast cancer, specifically through the NLRP3/caspase-1/GSDMD axis, thereby conferring antitumor activity (Yan et al., 2021). Additionally, LINC00969 has been shown to modulate the m6A methylation of NLRP3 through a METTL3–YTHDF2-dependent mechanism to influence its expression. This regulatory approach can suppress initiation of traditional pyroptosis signaling and may be linked to the development of chemoresistance in lung cancer cells (Dai et al., 2023). The lncRNA MALAT1 was shown to inhibit pyroptosis by modulating the miR-124/SIRT1 axis in cervical cancer (Liang et al., 2023); it can also prevent GSDMD-mediated pyroptosis in early metastatic cells by upregulating SERPINB6B expression, thus allowing the cells to evade CD8^+^ T-cell-mediated destruction and potentially reactivating dormant tumors (Kumar et al., 2024). Research efforts on the prognostic significance and impacts of pyroptosis-related long non-coding RNAs (PRlncRNAs) on immune responses against tumors in HCC remain limited.

In this study, we established a risk signature using eight PRlncRNAs and evaluated its effectiveness at predicting the prognosis and infiltration of immune cells in HCC patients using bioinformatic methods, with subsequent validation of our findings *in vitro*. We focused on the pyroptosis pathways and immune infiltration of LINC00942 and AC009283.1, which could guide us to predict survival and response to immunotherapy in HCC patients.

## 2 Materials and methods

### 2.1 Data acquisition

Our research involved RNA sequencing data as well as corresponding clinical details for a cohort of 373 patients diagnosed with HCC, along with lncRNA expression data retrieved from The Cancer Genome Atlas (TCGA) database (https://portal.gdc.cancer.gov/). The pyroptosis-related genes (PRGs) were sourced from the GeneCards database, and the disease-free survival (DFS) data were sourced from the cBioPortal database (https://www.cbioportal.org/).

### 2.2 Pathway enrichment analyses of the pyroptosis-related differentially expressed genes (PR-DEGs)

Functional assessments of the PR-DEGs were conducted using the Gene Ontology (GO) and Kyoto Encyclopedia of Genes and Genomes (KEGG) pathways.

### 2.3 Construction of a risk signature

The risk signature analysis was carried out using the R package “limma” to evaluate the relationship between the PR-DEGs and lncRNAs. In this process, the value of the absolute determination coefficient |R^2^| > 0.4 and *p* < 0.001 were included as the selection criteria. Next, univariate and multivariate Cox analyses were performed to construct the predictive signature of PRlncRNAs. When constructing this risk prognosis model, we calculated the area under the curve (AUC) value of the receiver operating characteristics (ROC) plot using the 95% confidence interval and used the mean value. The results showed that eight PRlncRNAs were included in the signature. The subsequent calculation of each patient’s risk score was based on the following formula:

$$\text{Risk score}=\sum_{i=1}^{n}\left(\text{Coe}f_{i}\times x_{i}\right),$$

where Coe f and x represent the regression coefficients and expression values of the PRlncRNAs, respectively.

### 2.4 Functional enrichment analysis of the PRlncRNA signature

The PRlncRNAs were divided into two categories according to their median risk scores. Gene set enrichment analysis (GSEA) was conducted using GSEA version 4.1.0 (http://www.broad.mit.edu/gsea/) with the conditions *p* < 0.05 and false discovery rate (FDR) <0.25 to determine the related signaling pathways. The level of immune cell infiltration was evaluated through single-sample GSEA (ssGSEA) and analysis of the gene set alterations.

### 2.5 Principal component analysis (PCA)

PCA was used to evaluate the reliability of the gene expression profiles across both the high-risk and low-risk cohorts. The “ggplot2” package of R software (version 3.3.3) was employed to analyze the clustering efficacy of the risk scores among the HCC patients.

### 2.6 Cell culture and transfection

MHCC-97H and Hep-3B cell lines were procured from American Type Culture Collection and incubated according to the provided instructions. Next, siRNAs (si-LINC00942 and si-AC009283.1) sourced from GenePharma (Shanghai, China) and Lipofectamine 2000 (Invitrogen, United States) were used for transfection. The specific siRNA sequences used are as follows: si-LINC00942: 5′-GUCUGCGGGAAACAGUACUTT-3′; si-AC009283.1: 5′-GCAAAUAGGUGUCUCAUAGCU-3′.

### 2.7 RNA extraction and quantitative real-time polymerase chain reaction (qRT-PCR)

The total RNA was extracted from the sample cells and HCC tissues using TRIzol reagent for subsequent reverse transcription and real-time quantitative PCR analysis. The RNA expression quantification was performed using the 2^−ΔΔCq^ method.

### 2.8 CCK-8, EdU, and colony formation assays

The cellular proliferation was assessed via the CCK-8 method. A total of 2,000 cells per well was cultured in a 96-well plate, with each well receiving 100 μL of the fully supplemented medium along with 10 μL of the CCK-8 reagent from TargetMol (United States). After an incubation period of 2 h, the optical density was determined at 450 nm. For the EdU assay, cells were seeded at the rate of 1 × 10^4^ per well in a 24-well plate and incubated with the EdU reagent at 37 °C for 2 h, following which they were fixed, permeabilized, and stained. The EdU-positive cells were then observed and counted using fluorescence microscopy. During the colony formation experiment, the cells were seeded at a density of 500 per well in a 6-well plate, and the cultures were permitted to proliferate over a period of 14 d. Then, the cells were fixed using a solution containing 4% paraformaldehyde and treated with a staining solution containing 0.5% crystal violet. The analysis was performed using ImageJ software.

### 2.9 Cell migration and invasion assays

In these assays, the cells were placed in the upper well of a chamber containing 200 μL of serum-deprived medium, while the lower well contained the medium enriched with all necessary supplements. Following a 24-h incubation period, the cells were fixed, stained, and enumerated using a microscope. Matrigel Matrix (cat no. 354234, Corning, United States) was applied to the upper compartment of the chamber and incubated for 48 h to measure the invasion capacity.

### 2.10 Western blotting and multiplex immunohistochemical (mIHC) analyses

Protein extraction was performed 72 h post-transfection, followed by separation using SDS-PAGE and transfer to a polyvinylidene fluoride membrane that was later incubated with primary and secondary antibodies for detection. The protein bands were observed using the ChemiDoc MP Imaging System. Paraffin-embedded HCC samples were deparaffinized using xylene and ethanol at different gradients. Following microwave-mediated antigen retrieval, the region of interest was outlined on each sample, and the sample underwent quenching and blocking procedures. The primary antibodies were incubated at room temperature for 1 h, followed by three washes with Tris-buffered saline with Tween-20 (TBST); then, the secondary antibodies were incubated at room temperature for 10–15 min. A fluorescence amplification reagent (Absin 4-Color IHC Kit, cat no. abs50028) was added for staining, incubated at room temperature for 10 min, and washed with TBST. Multiple antibodies were stained repeatedly, and the samples were finally counterstained with DAPI before scanning for visualization. The following antibodies were used in this stage: beta-tubulin (cat no. 10068-1-AP, 1:1,000, Proteintech, China); IL-18 (cat no. 10663-1-AP, 1:10,000); IL-1β (cat no. 16806-1-AP, 1:3,000); gasdermin D (GSDMD; cat no. 20770-1-AP, 1:2,000); caspase-1 (cat no. 22915-1-AP, 1:2,000/1:50, Proteintech, China); NLRP3 (cat no. 68102-1-Ig, 1:2,000/1:50, Proteintech, China); and CD3 (cat no. GB13440, 1:200, Servicebio, China).

### 2.11 Data analysis

The data were analyzed using R statistical software in conjunction with GraphPad Prism version 8. The expression levels of the PR-DEGs in the normal and HCC tissues were calculated through the Wilcoxon test. Both univariate and multivariate Cox regression analyses were carried out on the signature (binary variable), where the former was used to determine the relationship between the PRlncRNAs and overall survival (OS) of the HCC patients and the latter was used to construct the predictive signature with the selected lncRNAs. The log-rank test and Kaplan–Meier method were adopted to determine patient survival. The “survivalROC” package of R software was used to plot the ROC curves and calculate the AUC values. Wilcoxon signed-rank test and Spearman’s correlation analysis were adopted to evaluate the relevances between the immune cells and risk groups. The differences between the two different groups were detected through the Student’s t-test. All data points were presented as mean ± standard error of the mean (SEM) or standard deviation (SD), and the data were defined to be statistically significant at the *p* < 0.05 (*), *p* < 0.01 (**), and *p* < 0.005 (***) levels.

## 3 Results

### 3.1 GO and KEGG analyses of the PR-DEGs

**FIGURE 1:**
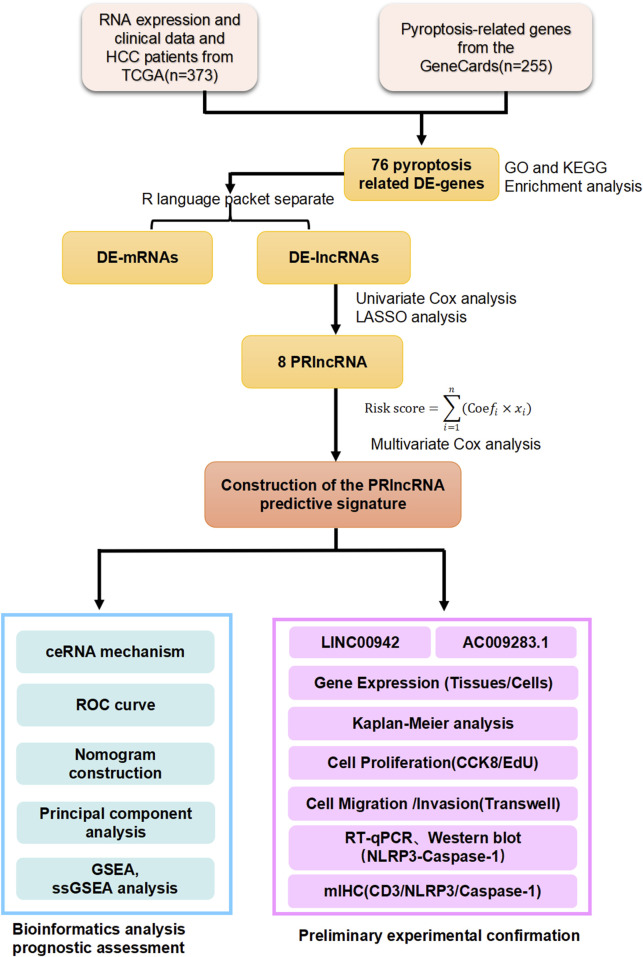
Schematic showing the experimental layout of the study.

Figure 1 provides a schematic representation of the experimental layout of the study. We identified 76 PR-DEGs from the GeneCards and TCGA databases, including 71 upregulated and 5 downregulated genes (Supplementary Figure S1A). The classical inflammasome assembly is provided by a Toll-like receptor (TLR) ligand, resulting in NF-κB-mediated transcriptional upregulation of the inflammasome components and pro-inflammatory cytokines like pro-IL-1β. NLRP3 is a common inflammasome composed of NLRP3, apoptosis-associated speck-like protein containing a CARD domain (ASC), and pro-caspase-1. Based on previous works and our specific interest in this field, we focused on the pyroptosis-related, TLR, and NF-κB signaling pathways via GO and KEGG analyses (Supplementary Figure S1B,C).

### 3.2 Risk model establishment based on PRlncRNAs

**FIGURE 2:**
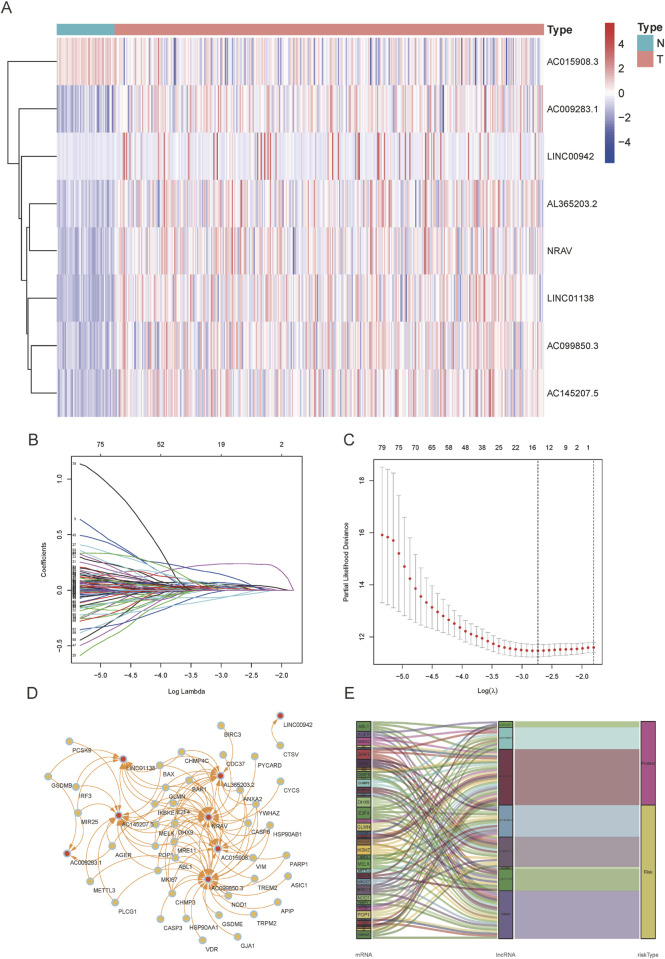
Expression levels and mRNA-lncRNA network of the eight pyroptosis-related long non-coding RNAs (PRlncRNAs) in the predictive signature. **(A)** Expression levels of the PRlncRNAs in normal and hepatocellular carcinoma (HCC) tissues. **(B, C)** Candidate PRlncRNAs from the univariate Cox regression analysis were filtered using the LASSO algorithm and coefficient profiles. **(D)** The mRNA–lncRNA co-expression network of the prognostic model for the eight PRlncRNAs. **(E)** Sankey diagram showing the prognosis based on the PRlncRNAs.

We established a risk model through Cox regression analysis on eight PRlncRNAs (AC015908.3, AC009283.1, LINC00942, AL365203.2, NRAV, AC099850.3, LINC01138, and AC145207.5) that have exhibited substantial correlations with the survival outcomes of individuals with HCC. Figure 2A illustrates the comparative expression profiles of the PRlncRNAs in HCC and normal tissues. The associations between these PRlncRNAs and prognosis were determined via LASSO regression (Figures 2B,C). Additionally, Figures 2D,E illustrate the mRNA-lncRNA co-expression network via the Cytoscape diagram and “ggalluvial” package in R software, highlighting the identification of LINC00942, AL365203.2, NRAV, LINC01138, and AC145207.5 as prognostic risk factors of HCC as well as AC015908.3, AC009283.1, and AC099850.3 as prognostic protective factors. The formula for calculating the risk score is as follows: risk score = (−0.415 × AC015908.3 expression) + (−1.166 × AC009283.1 expression) + (−0.467 × AC099850.3 expression) + (0.173 × LINC00942 expression) + (0.369 × AL365203.2 expression) + (0.632 × NRAV expression) + (0.784 × LINC01138 expression) + (1.334 × AC145207.5 expression).

### 3.3 Risk score as an independent prognostic indicator in HCC

**FIGURE 3:**
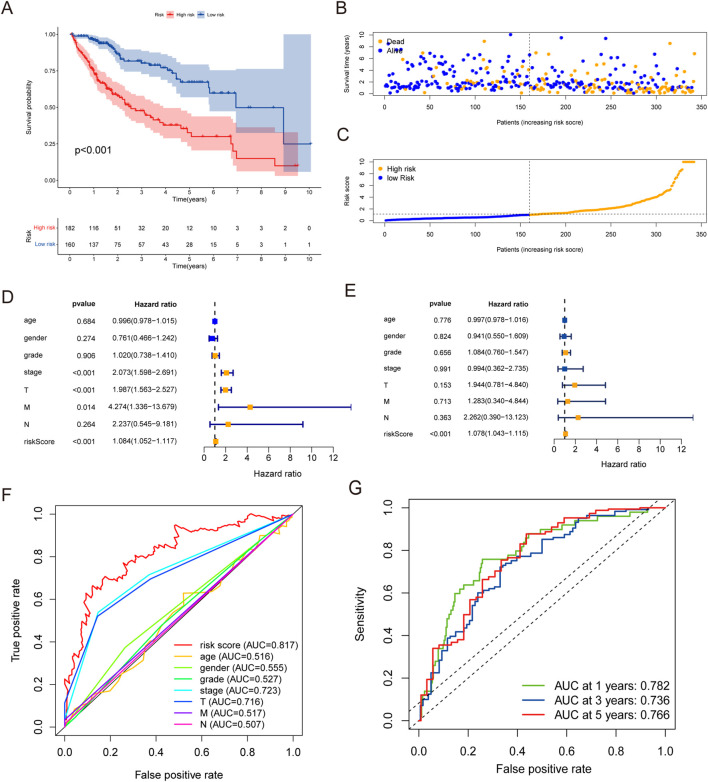
Prognostic value of the risk model based on the eight PRlncRNAs in the cancer genome atlas (TCGA) database. **(A)** Kaplan–Meier survival analysis of the high-risk and low-risk cohorts based on the risk model and median risk score. **(B)** Scatterplot based on the survival status of each sample, where the blue and orange dots represent survival and death, respectively. **(C)** Risk curve based on the risk score of each sample. **(D)** Forest plot for the univariate Cox regression analysis. **(E)** Forest plot for the multivariate Cox regression analysis. **(F)** Receiver operating characteristic (ROC) curves of the risk score and clinicopathological variables. **(G)** ROC curves and area under the curve (AUC) values at 1-, 3-, and 5-year survival for the predictive signature.

Stratifying the high-risk and low-risk cohorts based on the threshold median risk score showed that the high-risk cohort had poor OS (Figure 3A). An increase in the number of patient deaths was correlated with decreased survival time and increased risk score (Figures 3B,C). Significant differences in the clinicopathological indexes, including the T/N/M stage, overall stage, and grade, were assessed between the two cohorts (Supplementary Figure S2).

Cox regression analysis was used to assess various clinicopathological factors to validate the function of the risk score as a prognostic marker. The results of both univariate and multivariate Cox regression analyses confirmed the risk score as an independent prognostic factor (Figures 3D,E). The AUC of the risk score was calculated to be 0.764, which demonstrates superior predictive power compared to other prognostic factors (Figure 3F). The ROC analysis for predicting the 1-, 3-, and 5-year OS rates revealed values of 0.782, 0.736, and 0.766, respectively (Figure 3G). Furthermore, we constructed a nomogram integrating the risk score with additional clinicopathological factors to forecast the 1-, 3-, and 5-year OS probabilities (Supplementary Figure S3-1A). The predicted survival was highly consistent with the observed OS rate, affirming the reliability of the signature, as illustrated by the calibration curves (Supplementary Figures S3-1B–D) as well as the AUCs of the calibration curves in the nomogram and prognostic model (Supplementary Figures S3-2 ).

**FIGURE 4:**
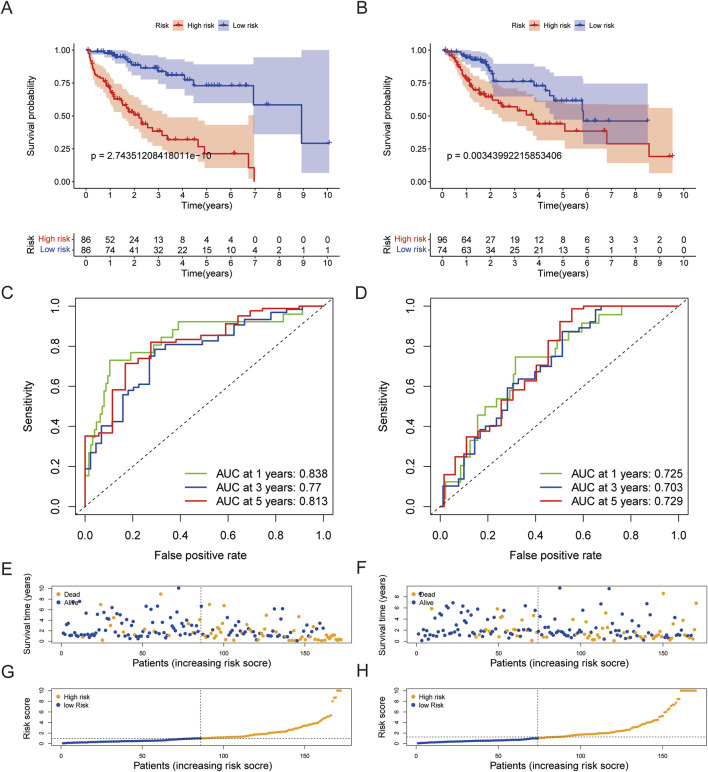
Verification of the prognostic model on the training and validation sets of TCGA database. **(A)** Kaplan–Meier survival analysis of the training set based on the high-risk and low-risk cohorts. **(B)** Kaplan–Meier survival analysis of the validation set based on the high-risk and low-risk cohorts. **(C, D)** ROC curves and AUCs at 1-, 3-, and 5-year survival for the training and validation sets. **(E, F)** Scatterplots based on the survival statuses of the samples from the training and validation sets, respectively. The blue and orange dots represent survival and death, respectively. **(G, H)** Risk curves based on the risk scores of the samples in the training and validation sets, respectively.

Additional confirmation was obtained using TCGA database from both the training and validation groups, involving OS analyses of both the high-risk and low-risk cohorts. The results consistently show poor OS for the high-risk cohort (Figures 4A,B). Furthermore, the 1-, 3-, and 5-year survival rates were significantly higher for the training cohort than the validation cohort (Figures 4C,D). Thus, a higher risk score was associated with greater patient mortality and poorer survival (Figures 4E–H).

We also explored the prognosis for various clinicopathological factors based on the risk score (Supplementary Figure S4), highlighting marked disparities in the OS between distinct high-risk and low-risk cohorts. The influence of gene expression on the clinicopathological factors (age, grade, gender, stage, and TNM stage) was assessed using six PRlncRNAs (AC015908.3, AC145207.5, AL365203.2, LINC00942, LINC01138, and NRAV) (Supplementary Figures S5A–O). Notable disparities were detected for the T and N stages based on the risk score (Supplementary Figures S5P–R). These results indicate that the PRlncRNA expression levels and risk score affect the clinical indexes and prognosis of HCC patients to a certain extent. Overall, the prognostic model demonstrates robust predictive capability based on various clinicopathological indexes; this underscores the risk score as an independent prognosis marker and the importance of accounting for individual patient factors when using the risk signature.

We also utilized the predictive model to analyze the DFS in HCC patients via the cBioPortal database, which matched with the OS results. The Kaplan–Meier analysis showed that the DFS was markedly lower in the high-risk cohort than the low-risk cohort (Supplementary Figures S6A–C). The 1-, 3-, and 5-year DFS rates are depicted in Supplementary Figures S6D–F and reinforce the model reliability in predicting the prognosis outcomes.

### 3.4 High-risk PRlncRNAs in tumor- and immunity-related pathway enrichment

**FIGURE 5:**
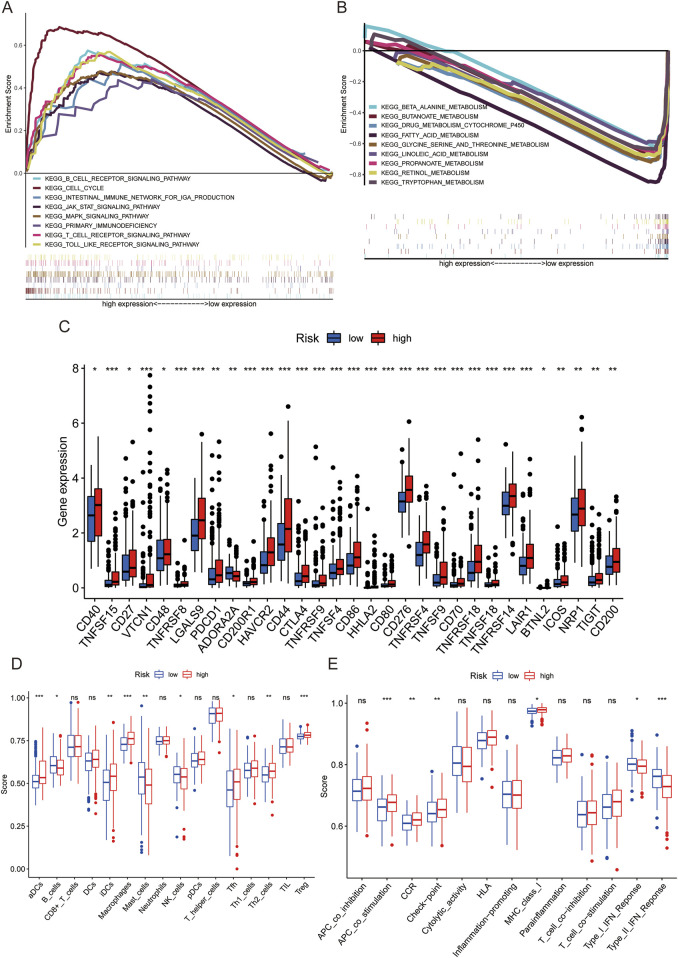
Gene set enrichment analysis (GSEA) and risk scores of the immune cells and immune-related pathways in the low-risk and high-risk cohorts. **(A)** Functional enrichment analysis of the high-risk PRlncRNAs. **(B)** KEGG pathway analysis of the low-risk PRlncRNAs. **(C)** Comparison of immune-checkpoint-blockade-related gene expression levels between the low-risk and high-risk cohorts. **(D)** Box plot of the infiltration levels of 16 immune cells in the high-risk and low-risk cohorts obtained by single-sample GSEA. **(E)** Correlations between the predictive signature and 13 immune-related pathways.

PCA was applied to the risk model to distinguish variations in the high-risk and low-risk cohorts. Supplementary Figures S7A–D, respectively, illustrate the 3D distribution of the entire genome, PRGs, PRlncRNAs, and risk genes. The results demonstrate that the low-risk and high-risk cohorts in the PRlncRNA set are separated into two parts, where the pyroptosis status of the patients in both cohorts are distinguishable. GSEA of the PRlncRNAs indicated that the high-risk cohort included the cell cycle, B-cell receptor signaling pathway, and T-cell receptor signaling pathway under KEGG enrichment (Figure 5A). Conversely, the metabolic pathways were found to be more enriched in the low-risk cohort (Figure 5B). This suggests that the PRlncRNAs in the high-risk cohort are largely linked to tumor and immunity pathways.

### 3.5 Low immune infiltration in the high-risk cohort of the prognostic model

The activation of immune checkpoints, immune cells, and cytokines is essential for immune responses. Notably, high levels of PDCD1, CTLA-4, and TIGIT were expressed within the high-risk cohort (Figure 5C). To investigate the link between the risk score and presence of immune cells, we analyzed the distributions of various immune cell subsets and immune-related pathways using ssGSEA. We discovered elevated levels of immune cell infiltration, including aDCs, iDCs, macrophages, Tfh, Th2, and Tregs, in the high-risk cohort. Conversely, the high-risk cohort exhibited reduced presence of B cells, mast cells, and natural killer (NK) cells (Figure 5D). The antigen-presenting cell co-stimulated pathway, CCR, checkpoint molecules, and major histocompatibility complex class I molecules showed increased activities in the high-risk cohort. However, individuals in the high-risk cohort exhibited reduced levels of both type-I and type-II interferons (Figure 5E).

### 3.6 Impacts of LINC00942 and AC009283.1 on pyroptosis pathways and immune infiltration

**FIGURE 6:**
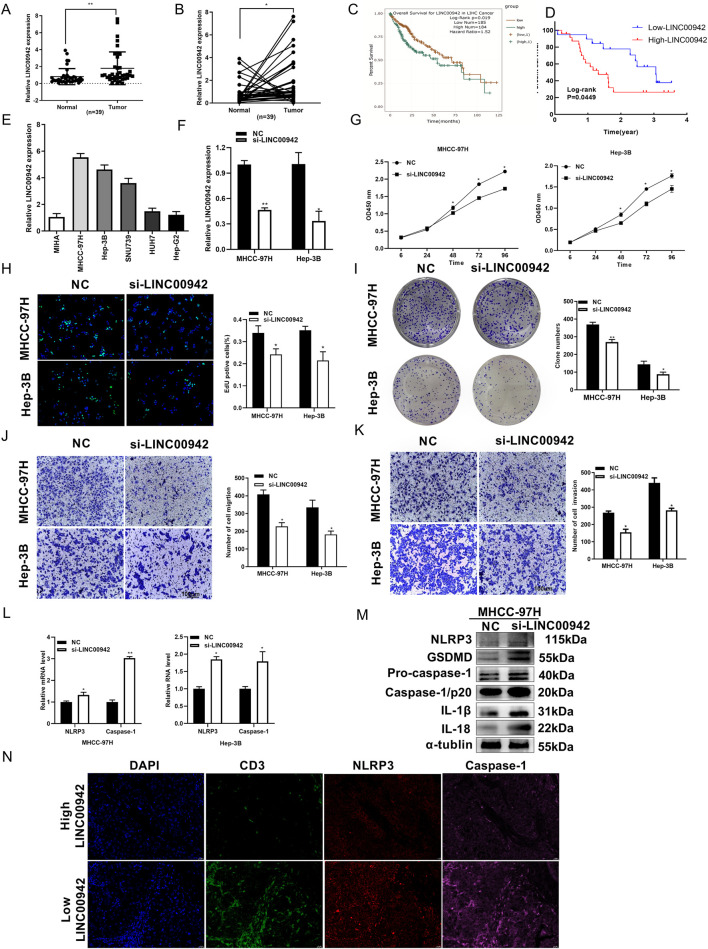
Effects of inhibiting the expression of LINC00942 on HCC cell proliferation progress. **(A, B)** Differential expressions of LINC00942 between cancer and non-carcinoma samples (n = 39). **(C, D)** Kaplan–Meier analysis of starBaseV3.0 (http://starbase.sysu.edu.cn/) and clinical samples from Xijing Hospital. **(E)** Comparison of normal liver and HCC cells for verifying LINC00942 expressions. **(F)** LINC00942 is downregulated in MHCC-97H and Hep-3B cells using siRNAs. **(G–I)** Proliferation of MHCC-97H and Hep-3B cells transfected with siRNAs against LINC00942 measured using the CCK-8, EdU, and colony formation assays, respectively. **(J, K)** Transwell assays with and without the Matrigel matrix to assess the migration and invasion abilities of si-LINC00942. **(L)** RNA expression levels of NLRP3/caspase-1 after knockdown of LINC00942. **(M)** Changes in the proteins after knockdown of LINC00942 assessed by Western blotting. **(N)** Co-localization of CD3 and classical markers of pyroptosis based on multiplex immunohistochemical assay. The paraffin-embedded HCC samples were immunofluorescently stained for NLRP3 (red), caspase-1 (pink), and CD3 (green). “ns” indicates not significant, *p < 0.05, **p < 0.01, and ***p < 0.005.

We selected the LNC000942 and AC009283.1 that, respectively, showed the highest and lowest risk coefficients for preliminary experimental validations. Here, LINC00942 exhibited a notably high expression level (Figures 6A,B), whereas AC009283.1 exhibited low expression level in the HCC samples (Supplementary Figures S8A,B). Kaplan–Meier analysis results based on the ENCORI starBaseV3.0 (https://rnasysu.com/encori/panCancer.php) and clinical samples from Xijing Hospital indicated that high expression of LINC00942 is correlated with poor survival for HCC patients (Figures 6C,D) and that its expression is significantly associated with tumor size (Table 1). Conversely, low expression level of AC009283.1 is linked to adverse prognosis (Supplementary Figures S8C,D) and correlated with a higher maximal tumor number (Supplementary Table S1).

**TABLE 1:** Correlations between LINC00942 expression level and some clinicopathological characteristics in hepatocellular carcinoma.

Parameter	LINC00942 expression level		$\chi^{2}$	*p*-value
	Low (n = 20)	High (n = 24)		
Gender			0.023^a^	ns
Male	17	20		
Female	3	4		
Age (years)			0.642^a^	0.423
<50	6	10		
≥50	14	14		
Maximal tumor size			4.130^a^	0.042*
<5 cm	10	5		
≥5 cm	10	19		
Tumor number			0.489^a^	0.484
Single	16	18		
Multiple	4	6		
TNM stage			0.095^a^	0.757
I–II	15	17		
III–IV	5	7		
HBeAg			0.466^a^	0.710
Negative	15	20		
Positive	5	4		
AFP			0.210^a^	0.647
<400 ng/mL	12	16		
≥400 ng/mL	8	8		
Differentiation			0.482^a^	0.786
Well	3	4		
Moderate	15	16		
Poor	2	4		

Statistical analysis was performed using Pearson’s $\chi^{2}$ test.

^a^ Fisher’s exact test. Statistical significance: “ns” means not significant, *p < 0.05, **p < 0.01, and ***p < 0.005.

To further explore the roles of LINC00942 and AC009283.1, we used the MHCC-97H and Hep-3B cell lines (Figure 6E; Supplementary Figure S8E). Transfection with siRNA resulted in significant downregulation of both LINC00942 and AC009283.1 (Figure 6F; Supplementary Figure S8F). Depletion of LINC00942 resulted in suppression of cellular proliferation, migration, and invasive capabilities, which were confirmed through CCK-8, EdU, colony formation, and Transwell assays (Figures 6G–K). Furthermore, suppression of LINC00942 led to elevated expressions of NLRP3 and caspase-1 at the RNA level (Figure 6L) as well as GSDMD, NLRP3, caspase-1, IL-1β, and IL-18 in the MHCC-97H cells at the protein level (Figure 6M).

We performed mIHC assays on four clinical HCC specimens from Xijing Hospital, which showed a correlation between LINC00942 expression and pyroptosis/immune markers. A higher expression of LINC00942 corresponded with lower concentrations of NLRP3/caspase-1 and CD3 (Figure 6N), which could inhibit pyroptosis and immune infiltration in the tumor cells. In contrast, downregulation of AC009283.1 promoted proliferation and metastasis of HCC, which could inhibit pyroptosis and immune infiltration *in vitro* (Supplementary Figures S8G–N).

## 4 Discussion

As a new form of inflammatory-mediated programmed cell death, studies have revealed that pyroptosis is closely associated with progression of malignancies (Rao et al., 2022). Pyroptosis is usually triggered by inflammasomes and executed by gasdermin proteins, where NLRP3 inflammasome formation is a key event in the activation of the classical pyroptosis pathway. The NLRP3 inflammasome converts pro-caspase-1 to cleaved caspase-1 and then cleaves GSDMD, leading to pyroptosis. Recent studies have shown that lncRNAs substantially influence pyroptosis regulation with inflammatory or immune responses via the NLRP3/caspase-1/GSDMD pathway (Liu et al., 2024) and that PRlncRNAs may be involved in tumor progression and antitumor immune response promotion (Kumar et al., 2024).

In this study, we identified eight PRlncRNAs to establish a risk signature as well as investigate its prognostic value and immune infiltration in HCC patients. Survival analysis in terms of the OS and DFS based on the Kaplan–Meier method revealed that HCC patients classified under the high-risk cohort experienced poorer survival than those in the low-risk cohort. Thus, the risk score is effective for assessing the prognosis of HCC patients than various clinical characteristics, including age, stage, and TNM classification. The findings suggest that the risk score possesses a certain degree of precision as a prognostic factor, which is crucial for assessing the prognosis of HCC patients and distinguishing the population that benefits from cancer treatment.

The signature based on the PRlncRNAs not only serves as an independent prognostic factor but also predicts immune infiltration within the tumor microenvironment (TME) (Wang L. et al., 2024). The TME is significantly composed of immune cells that contribute uniquely to the immune system’s fight against tumors. For example, therapy triggering pyroptosis was shown to boost the penetration of CD4^+^ T cells and CD8^+^ T cells along with NK cells in the TME while causing decreases in the numbers of monocytes, neutrophils, and myeloid-derived suppressor cells in breast tumors (Wang et al., 2020). Our risk signature reveals that reducing the counts of NK and B cells while enhancing the counts of macrophages, Tregs, and iDCs in the high-risk cohort may negatively regulate inflammatory/immune responses and result in HCC progression. Fewer numbers of NK cells may reduce recognition of the surface molecules of tumor cells as well as weaken cytotoxicity through reduced release of perforins and granzymes to prevent tumor cell death (Sankar et al., 2022). Increased number of macrophages in HCC can promote differentiation of naive T cells into Tregs through cytokine secretion as well as recruitment of mature Tregs to the tumor sites (Vilbois et al., 2024). Additionally, Tregs suppress immune activity by curbing the activation of immune cells, thus averting excessive immune reaction within HCC (Wang H. et al., 2024). Although iDCs are adept at capturing antigens, their limited capacity to activate T cells may impede antitumor immune responses in the high-risk cohort.

Pyroptosis is also linked to immune checkpoints. When tumor cells undergoing pyroptosis release tumor antigens to strengthen adaptive immune responses, the efficacies of the ICIs are boosted (Jia et al., 2023). Individuals in the high-risk cohort showed increased expressions of genes associated with immune checkpoints, including PDCD1 (PD-1), CTLA-4, and TIGIT, which could potentially impact immune reactions in the TME. T cells expressing inhibitory receptors like PD-1 may lead to T cell exhaustion, thereby promoting immune evasion (Zeng et al., 2020). PD-1 may protect against autoimmunity by promoting apoptosis of antigen-specific T cells, while the TIGIT receptor is capable of suppressing the functionality of NK cells and diminishing the reactivity of CD8^+^ T cells, further underscoring the challenges in tumor regression (Banta et al., 2022). Increased expressions of these inhibitory checkpoints in the high-risk cohort suggest that PRlncRNAs could be valuable for assessing immune status within the TME and supporting choices in cancer treatment.

In addition to activation of immune cells and inhibition of immune checkpoints influencing the TME, the release of cytokines exerts a certain regulatory effect on the immunosuppressive microenvironment (Li et al., 2023). Among the biological processes of the GO enrichment analysis, we found that PR-DEGs (including the eight PRlncRNAs investigated herein) were predominantly distributed in the positive regulation of cytokine production, regulation of responses to cytokine stimulus, and regulation of cytokine-mediated signaling pathways. There are several types of chemokines and their corresponding receptors that have both tumor-suppressing and tumor-promoting activities. For example, CCR4 can recruit Tregs in the TME, and CCR6 can promote proliferation of Tregs *in situ* in the TME (Kohli et al., 2022); CCR7 can mediate migration of cDC1 and cDC2 cells from tumors to tumor-draining lymph nodes, thereby initiating the effector function of T cells (Brandum et al., 2021). We propose a preliminary guess that the increase in CCR in the high-risk cohort may affect the occurrences of these chemokines. Additionally, reductions in type-I and type-II interferon responses were observed in the high-risk cohort. This decreased response to interferons may contribute to uncontrolled cellular proliferation, heightened immune checkpoint tolerance, and reduced sensitivity of the checkpoint proteins to inhibitors, which further affect tumor immune escape and tolerance. These findings suggest that the high-risk cohort commonly experiences a low-level activation of immune responses, highlighting the potential for tailored immunotherapy approaches in HCC and the importance of seeking new immunotherapy strategies.

In the risk signature proposed herein, LINC00942, AL365203.2, NRAV, LINC01138, and AC145207.5 are identified as HCC prognostic risk factors, while AC015908.3, AC009283.1, and AC099850.3 are found to be prognostic protective factors. LINC00942 is an oncogene that has been previously identified in HCC and other cancers (Jin et al., 2024; Sun et al., 2022; Wang et al., 2021). Studies have reported that LINC00942 has significantly elevated expression in HCC and impedes the progression of ferroptosis to promote immunosuppression of Tregs (Jin et al., 2024); its high expression level not only inhibits apoptosis and autophagy but also promotes stemness and chemoresistance in germinal center cells (Zhu et al., 2022). LINC00942 had been previously reported to regulate ferroptosis, apoptosis, and autophagy. The present research further investigated its potential role in HCC, particularly in relation to pyroptosis and immune infiltration. LINC00942 has high expression levels and promotes the progression of HCC. We preliminarily speculated that knockdown of LINC00942 could trigger the classical NLRP3/caspase-1/GSDMD pyroptosis signaling pathway, thereby influencing the secretion of cytokines IL-1β and IL-18. In contrast, AC009283.1 was recognized as a tumor-suppressor gene that inhibits progression of HCC. Its high expression is associated with antitumor effects as well as increased pyroptosis and immune infiltration. The present study indicates that LINC00942 and AC009283.1 are promising prognostic predictors that may aid in evaluating the immune status in HCC patients to inform tailored immunotherapy approaches. Beyond mere destruction of cancer cells, triggering of pyroptosis can counteract immune suppression and elicit robust antitumor immune responses to offer significant options for cancer treatment (Tan et al., 2021).

Practical application of the prognostic risk model based on PRlncRNAs in clinical practice is mainly aimed at prognosis prediction, treatment stratification, and immunotherapy guidance, especially for stratifying patients treated with ICIs. The PRlncRNA-based risk model can be used to assess the immune infiltration status of the TME, where the high-risk cohort shows decreased infiltration of CD8^+^ T cells and increased expression of immune checkpoints (such as PD-L1 and CTLA-4) that are suitable for ICI treatment. The low-risk cohort shows poor immunosuppression, high infiltration of T cells, and may require combined pyroptosis induction therapy to enhance the efficacies of the ICIs. The PRlncRNA model can help distinguish between “immune hot” and “immune cold” tumors, and the low-risk cohort that has more T cell infiltration may benefit more from PD-1/PD-L1 inhibitors. Studies have shown that mRNA-LNP-mediated GSDMB pyroptosis can activate the TME, transform “cold tumors” to “hot tumors”, significantly improve the efficacies of PD-1 inhibitors, and even achieve complete tumor regression in mouse models. The risk score guides the combination regimen in the high-risk cohort of HCC patients (with low pyroptosis efficiency), who may require pyroptosis-inducing agents (e.g., GSDMB mRNA) and ICIs, whereas the low-risk cohort of HCC patients (with high pyroptosis efficiency) may be treated sufficiently with ICIs alone. The PRlncRNA risk model can accurately predict patient survival and optimize immunotherapy stratification as well as improve the efficacy of treating “cold tumors” through pyroptosis-induced ICI strategies in the future. However, its clinical implementation needs further standard and cost optimizations even given the significant conversion potential.

Although we successfully constructed a PRlncRNA risk model in this study to predict the prognosis and immune infiltration status of HCC patients, the following challenges need to be addressed. First, TCGA database is mainly composed of retrospective data, and there is an inherent bias in these data owing to the high proportion of early-stage patients and selection bias in the treatment heterogeneity. There is also a lack of multicenter external validation cohorts, and the universality needs to be verified through prospective studies. Second, standard TCGA data, such as RNA sequencing results, are derived from whole tumor tissues composed of a mixture of various cell types, including tumor cells, immune cells, stromal cells, and normal epithelial cells. Unfortunately, it is not feasible to directly isolate signals from pure single-cell populations (e.g., exclusively CD8 T cells) from the raw TCGA data; hence, existing single-cell sequencing data need to be learned for screening HCC prognosis (Ge et al., 2024). CIBERSORT, xCell, and single-cell RNA sequencing data must be assessed to address disadvantages and accurately identify immune cells or markers. Third, PRlncRNA as a biomarker for HCC prognosis prediction offers unique advantages over traditional indicators like AFP and TNM stage. It is inevitable that PRlncRNAs may also have limitations in predicting the prognosis of patients with HCC. While AFP detection is standard and inexpensive, the detection of PRlncRNAs necessitates techniques like RT-PCR or sequencing, both of which are characterized by high technical thresholds and high costs. Finally, the present study preliminarily shows that PRlncRNAs may influence the regulation of pyroptosis and T cell immune infiltration; however, additional investigative efforts are required to thoroughly clarify the underlying mechanisms of how PRlncRNAs influence antitumor immune processes in HCC. In the future, organoid models and tumor cells with immune cell co-cultures could be considered to evaluate the effects of PRlncRNAs on the immune microenvironment in HCC.

## 5 Conclusion

The proposed PRlncRNA risk model for HCC patients proves that the risk score is an independent prognostic indicator with greater predictive accuracy than conventional clinical factors. Our results show that the risk score as well as expression levels of LINC00942 and AC009283.1 could play significant roles in forecasting patient prognosis, assessing the impacts of immune infiltration on HCC, and possibly informing therapeutic decisions in clinical practice.

## Data Availability

The datasets presented in this study can be found in online repositories. The names of the repositories and accession numbers can be found in the article/Supplementary Material.
