# Supplementary material for: Identifying pyroptosis-related lncRNAs to predict prognosis and immune regulation in hepatocellular carcinoma
Source: Front Mol Biosci. 2026 Feb 25;13:1714966. doi: 10.3389/fmolb.2026.1714966 (PMC12975465; doi:10.3389/fmolb.2026.1714966)
Supplement: Supplementary file 1 [file Supplementaryfile1.docx]

Supplementary Material

# Supplementary Figures and Tables

#
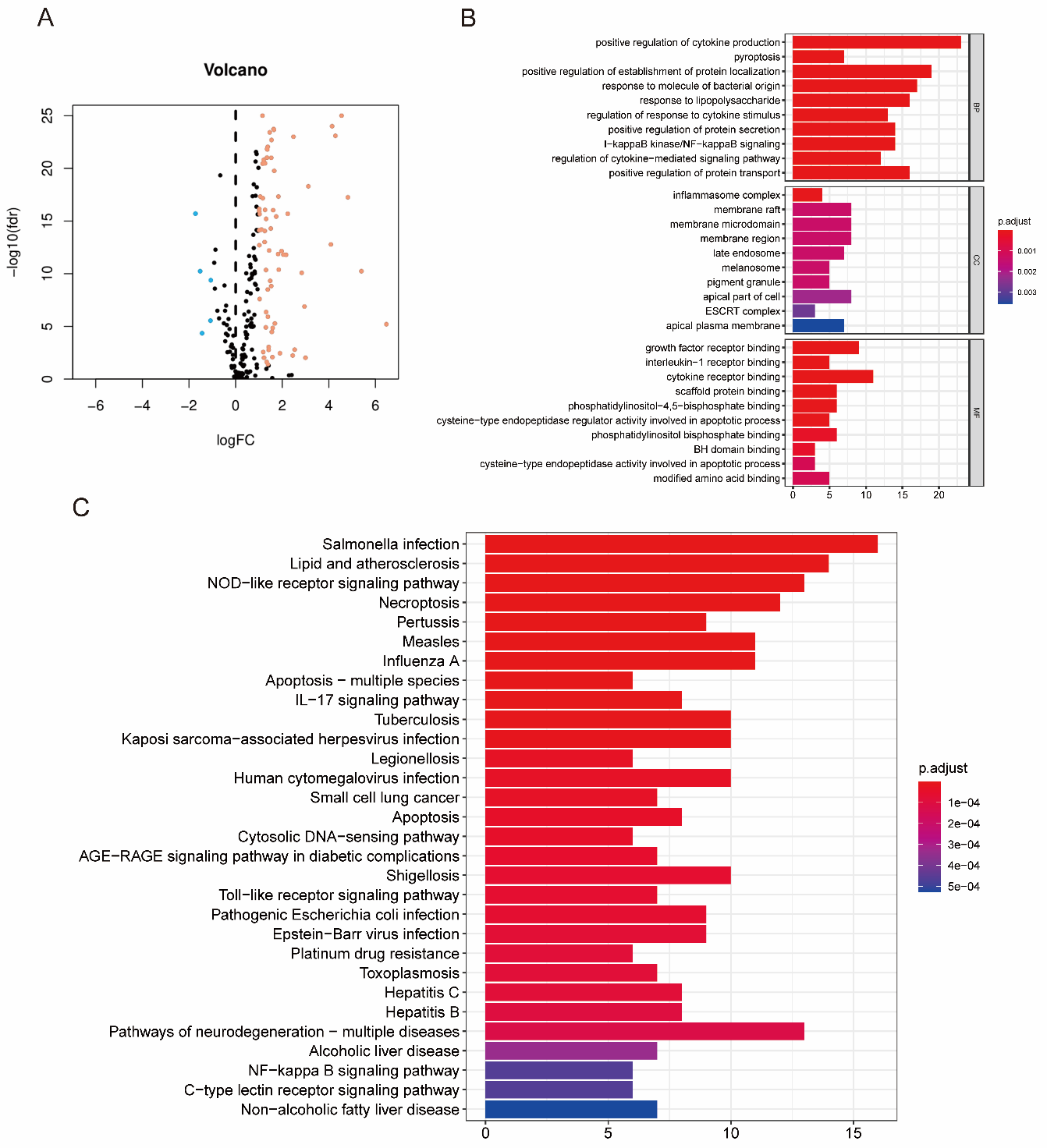


# Figure S1. Analysis of PR-DEGs.

# (A)The volcano plot showed that 71 differentially expressed genes were up-regulated (orange) and 5 down-regulated (blue) in HCC. (Fold change >4, p = 0.001). (B) GO analysis of DEGs. (C) KEGG analysis of DEGs.


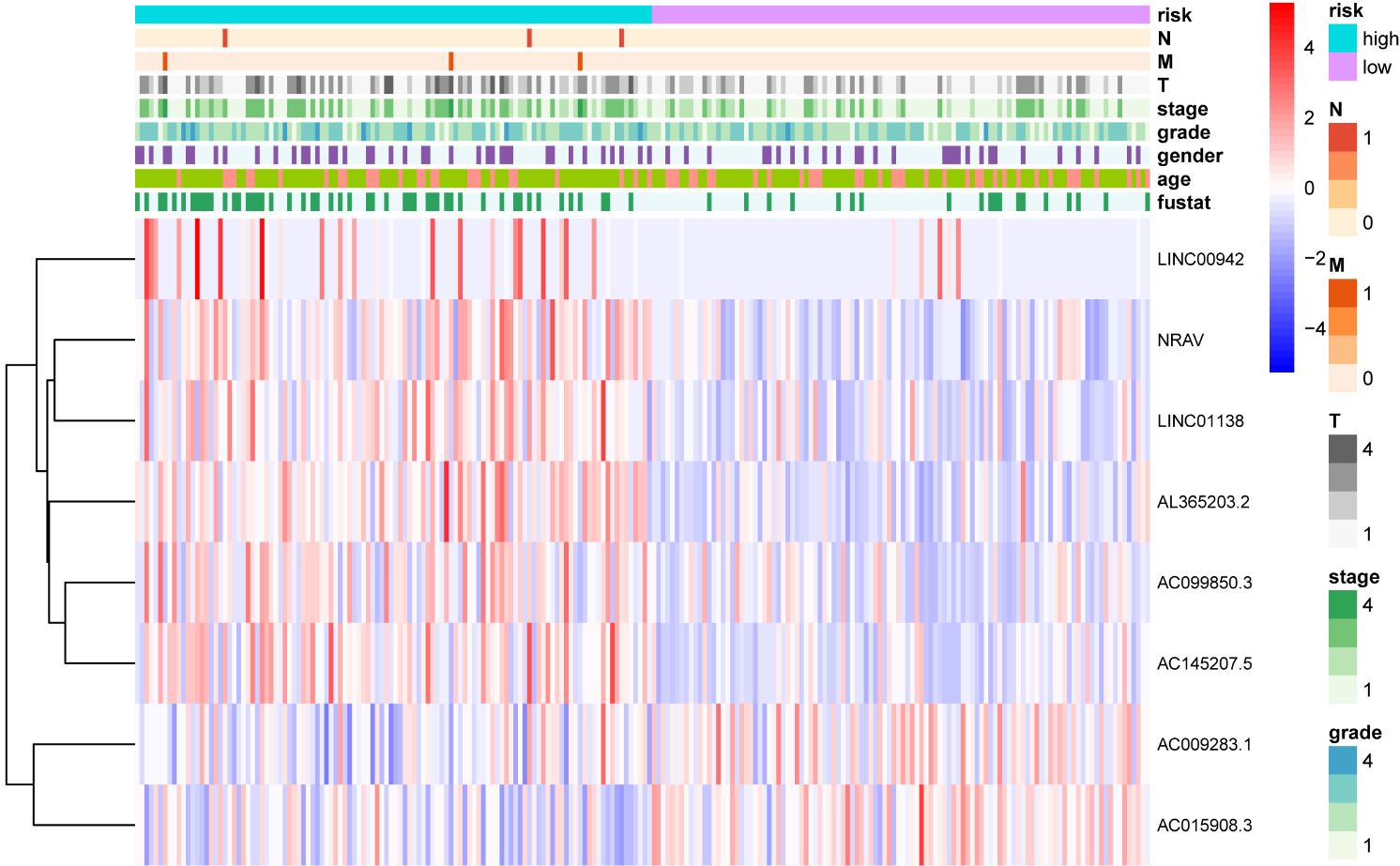


# Figure S2 The heatmap displayed the clinicopathological variables of 8 PRlncRNAs in the high-risk and low-risk cohorts.


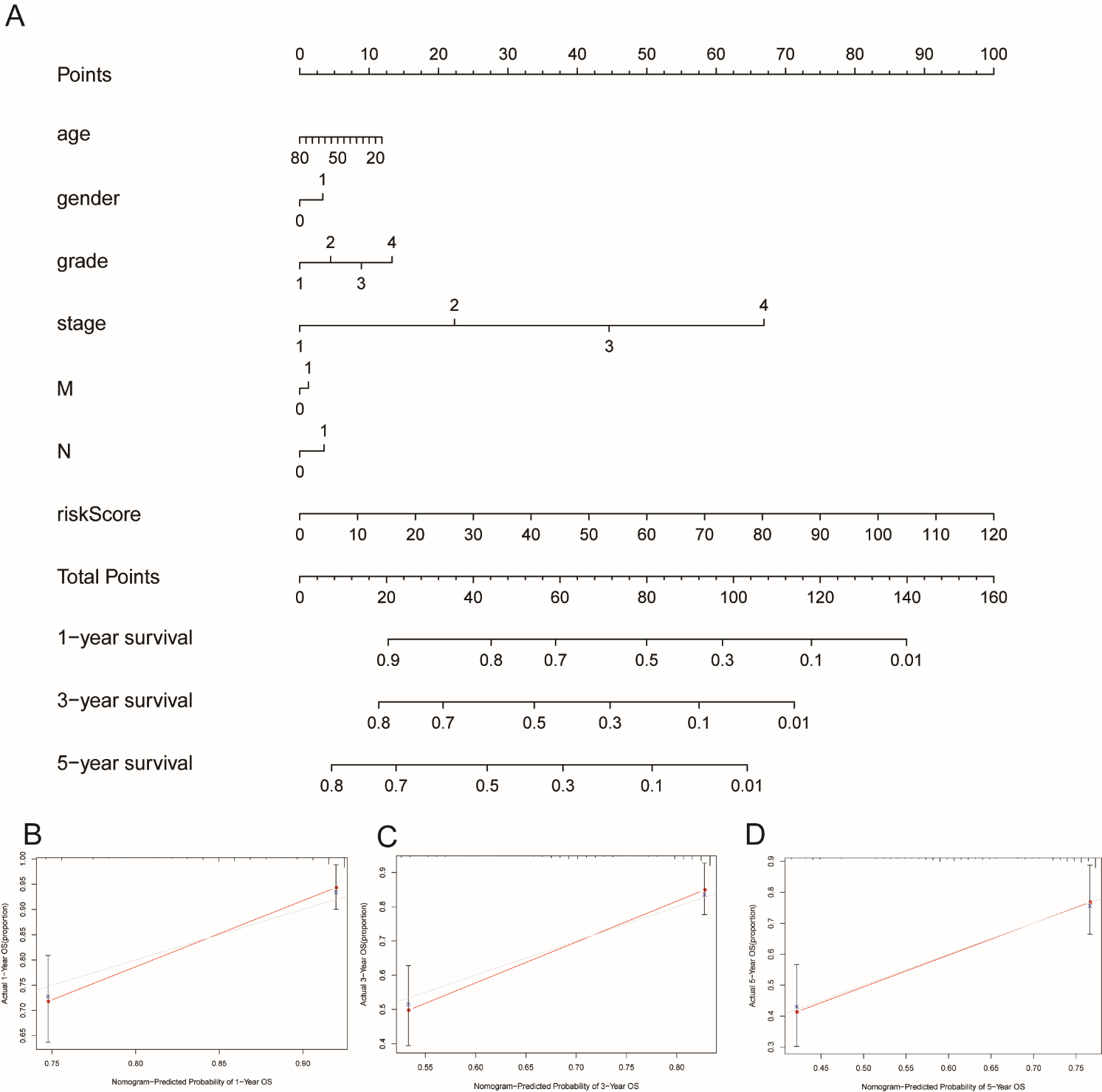


# Figure S3-1. Construction and verification of the nomogram.

# A nomogram combining clinicopathological variables and risk score predicts 1-, 3-, and 5-years OS of HCC patients. (B-D) The calibration curves test consistency between the actual OS rates and the predicted survival rates at 1-, 3-, and 5-years.


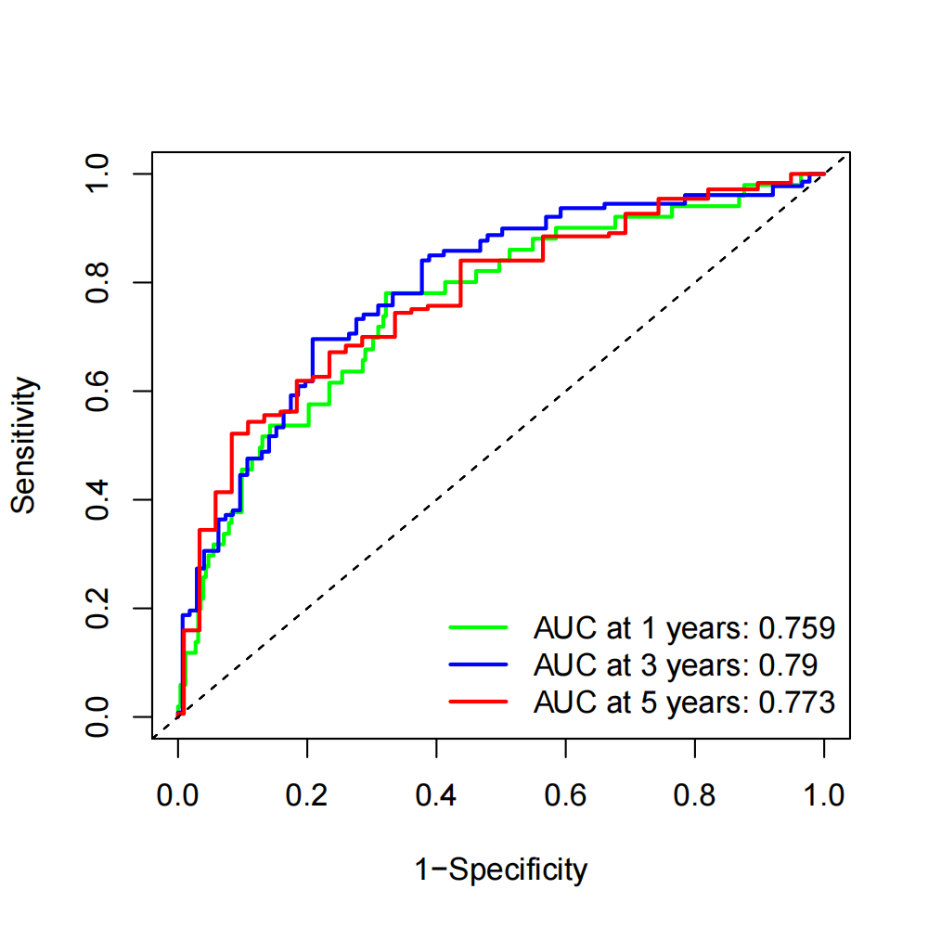


# Figure S3-2. The AUC of calibration curves in nomogram and prognostic model.


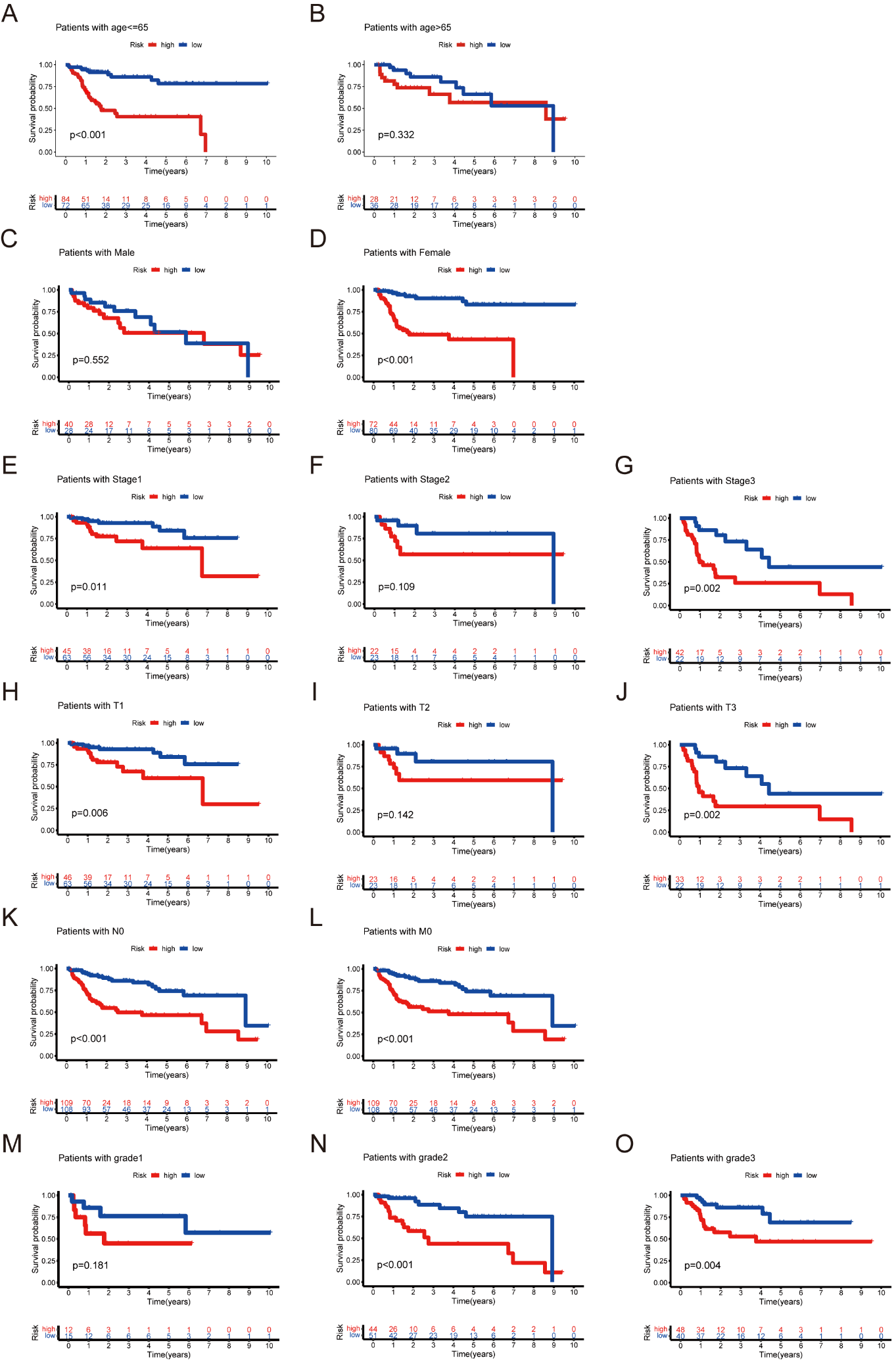


# Figure S4. Kaplan-Meier survival curves of HCC patients classified according to different clinicopathological variables in high- and low-risk cohorts.

# (A-B) Age. (C-D) Sex. (E-G) Stage. (H-J) T stage. (K) M stage. (L) N stage. (M-O) Grade.


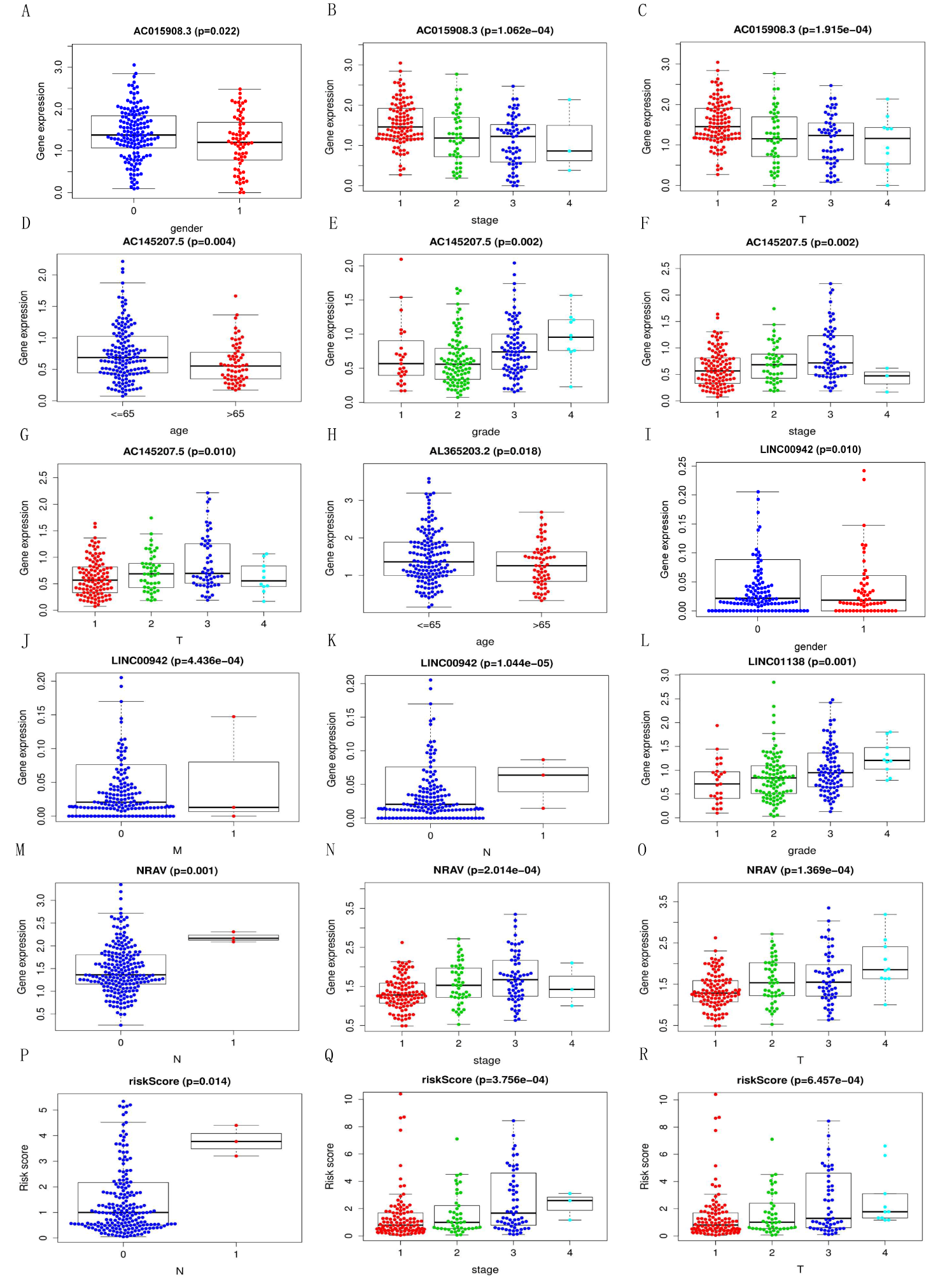


# Figure S5. Clinical features and risk score analysis of PRlncRNAs.

# (A-C) AC015908.3 expression analysis in gender, stage, T stage. (D-G) AC145207.5 expression analysis in age, grade, stage, T stage. (H) AL365203.2 expression analysis in age. (I-K) LINC00942 expression analysis in gender, M stage, N stage. (L) LINC01138 expression analysis in grade. (M-O) NRAV expression analysis in N stage, stage, T stage. (P-R) Risk score in T stage, stage and N stage.


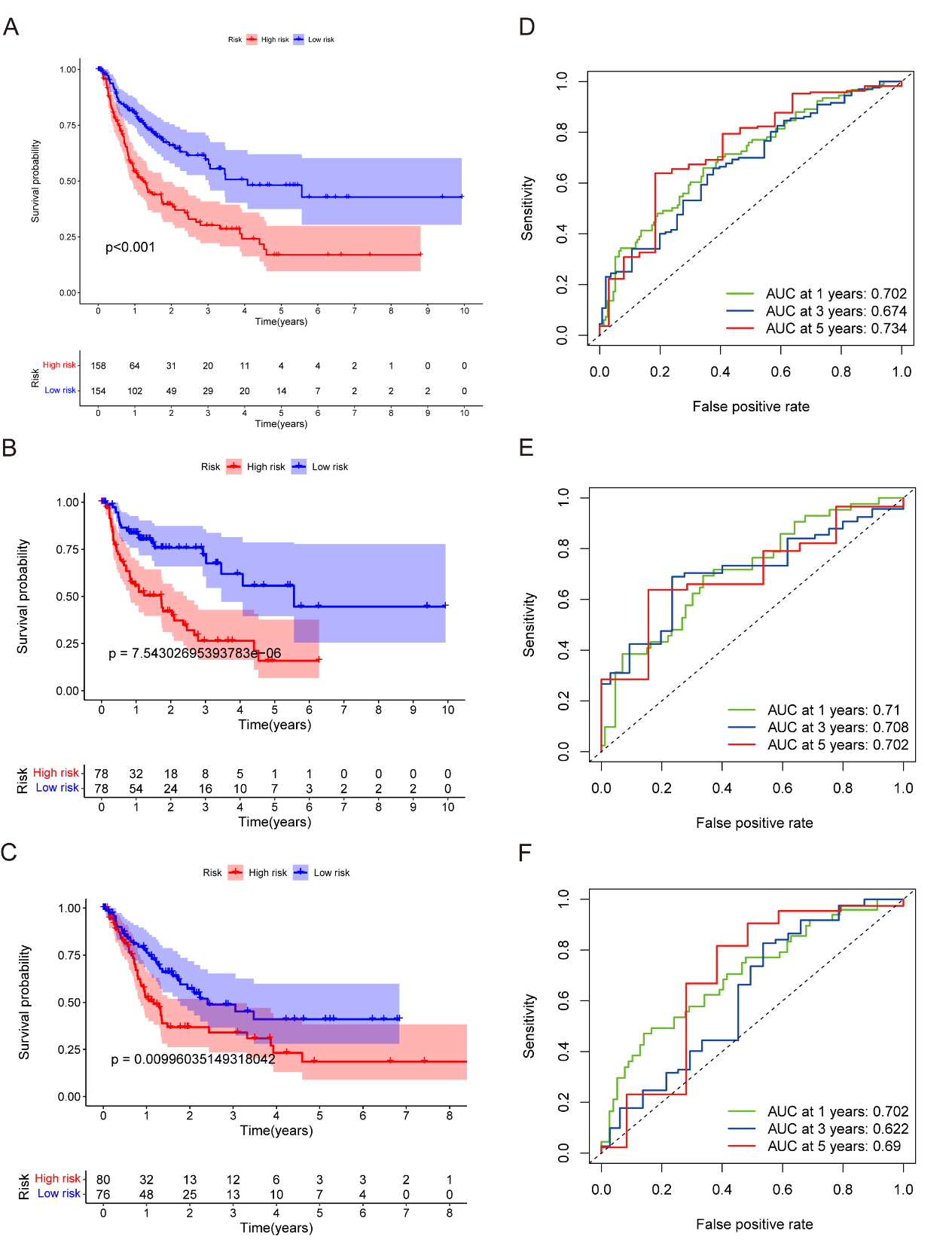


# Figure S6. Evaluation of the predictive value of the PRlncRNA signature for DFS.

# Kaplan-Meier survival curve in the entire dataset. (B) Kaplan-Meier survival curve in the first internal set. (C) Kaplan-Meier survival curve in the second internal set. (D) ROC curve and AUCs at 1-, 3- and 5-years survival in the entire dataset. (E-F) ROC curve and AUCs at 1-, 3- and 5-years survival in the first and second internal set.


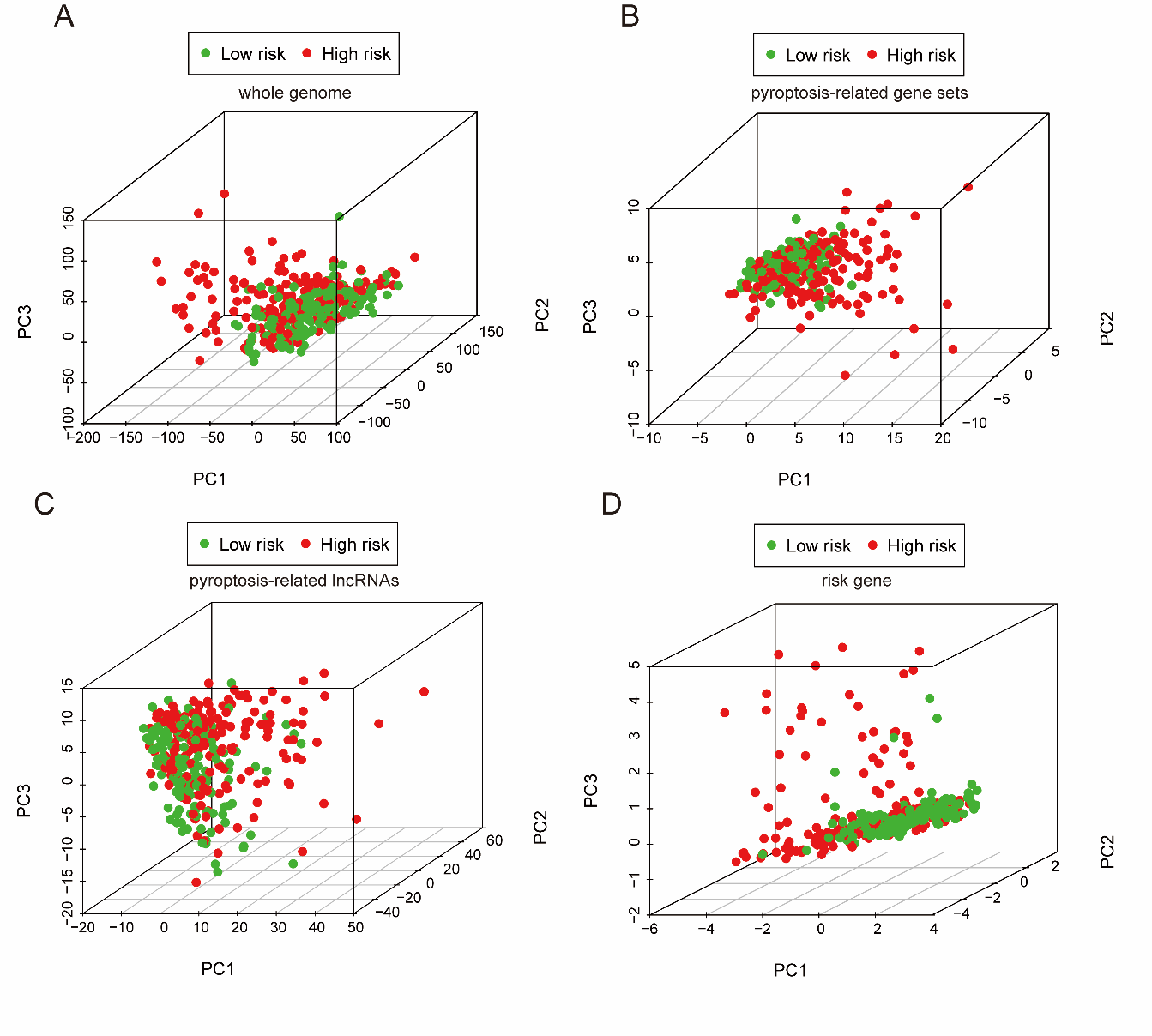


# Figure S7. PCA between the low-risk and high-risk cohorts gene expression profiles.

# (A)Distribution of genome in risk cohort. (B) Distribution of PRGs sets in risk cohort. (C) Distribution of PRlncRNAs in risk cohort. (D) Distribution of risk gene in risk cohort.


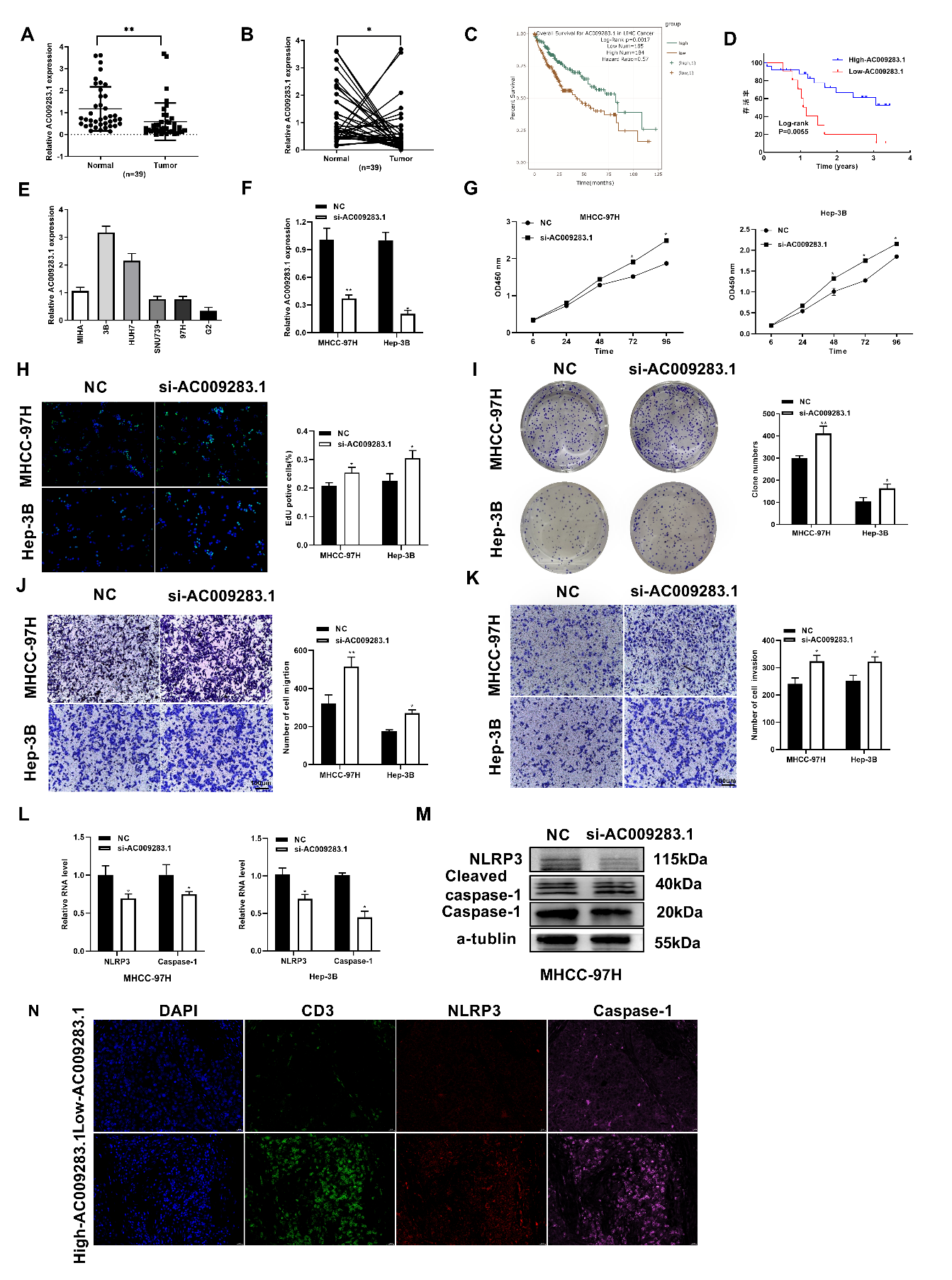


# Figure S8. Effects of inhibiting the expression of AC009283.1 on HCC cell proliferation progress.

# (A-B) Differential expression of AC009283.1 between cancer and non-carcinoma samples (n=39). (C-D) Kaplan-Meier analysis of starBaseV3.0 (http://starbase.sysu.edu.cn/) and clinical samples from Xijing Hospital. (E) Normal liver cell and HCC cells to verify the AC009283.1 expression. (F) AC009283.1 was downregulated in MHCC-97H and Hep-3B cells using siRNAs. (G-I) The proliferation of MHCC-97H and Hep-3B cells transfected with siRNA against AC009283.1 were measured using CCK8 assays, Edu assays and colony formation assays. (J-K) Transwell with/without the Matrigel Matrix to test the migration and invasion ability of si-AC009283.1. (L) The RNA expression levels of NLRP3/Caspase-1 after the knockdown of AC009283.1. (M) The protein levels of NLRP3/Caspase-1 after the knockdown of AC009283.1. (N) Co-localization of CD3 and pyroptosis classical markers via mIHC. Paraffin-embedded HCC samples underwent immunofluorescent staining for NLRP3 (Red), Caspase-1 (Pink), and CD3 (Green).

**Supplementary Table 1**

**Table 1** Correlation between AC009283.1 expression and clinicopathological characteristics in HCC

|  | **AC009283.1 expression levels** | | | $\boldsymbol{\chi}^{\boldsymbol{2}}$ | **P value** |
| --- | --- | --- | --- | --- | --- |
|  | | **Low(n=11)** | **High(n=25)** |  |  |
| **Gender** | |  | | 0.305 ^a^ | ns |
| Male | | 10 | 21 |  | |
| Female | | 1 | 4 |  |  |
| **Age** | |  | | 2.207 ^a^ | 0.259 |
| <50 | | 2 | 11 |  | |
| >=50 | | 9 | 14 |  |  |
| **Maximal tumor size** | |  | | 0.582 ^a^ | 0.454 |
| <5cm | | 4 | 6 |  | |
| >=5cm | | 7 | 19 |  |  |
| **Tumor number** | |  | | 5.657 ^a^ | **0.039*** |
| Single | | 5 | 21 |  | |
| Multiple | | 6 | 4 |  |  |
| **TNM stage** | |  | | 1.657^a^ | 0.252 |
| I-II | | 6 | 19 |  | |
| III-IV | | 5 | 6 |  |  |
| **HBeAg** | |  | | 3.168^a^ | 0.148 |
| Negative | | 11 | 23 |  | |
| Positive | | 0 | 2 |  |  |
| **AFP** | |  | | 1.6571^a^ | 0.252 |
| <400ng/ml | | 6 | 19 |  | |
| >=400ng/ml | | 5 | 6 |  |  |
| **Differentiation** | |  | | 0.309 ^a^ | 0.857 |
| Well | | 2 | 4 |  | |
| Moderately | | 8 | 17 |  |  |
| Poorly | | 1 | 4 |  |  |

Statistical analysis was performed by Pearson’s $\boldsymbol{\chi}^{\mathbf{2}}$ test. ^a^ Fisher’s exact test. *Statistically difference.

ns represented for not significant, p <0.05 represented by *, p<0.01 represented by **, and p<0.005 represented by ***.
